# Successful MDR-TB treatment regimens including Amikacin are associated with high rates of hearing loss

**DOI:** 10.1186/1471-2334-14-542

**Published:** 2014-10-09

**Authors:** Chawangwa Modongo, Rafal S Sobota, Boikobo Kesenogile, Ronald Ncube, Giorgio Sirugo, Scott M Williams, Nicola M Zetola

**Affiliations:** Division of Infectious Diseases, University of Pennsylvania, Philadelphia, Pennsylvania USA; Botswana-University of Pennsylvania Partnership, 214 Independence Avenue, Gaborone, Botswana; Department of Medicine, University of Botswana, Gaborone, Botswana; Department of Genetics, Geisel School of Medicine, Dartmouth College, Hanover, NH USA; Princess Marina Referral Hospital, Gaborone, Botswana; National Tuberculosis Program, Ministry of Health, Gaborone, Botswana; Centro di Ricerca, Ospedale San Pietro Fatebenefratelli, Rome, Italy

**Keywords:** Hearing loss, Aminoglycosides, Amikacin, Multi-drug resistant tuberculosis, HIV

## Abstract

**Background:**

Aminoglycosides are a critical component of multidrug-resistant tuberculosis (MDR-TB) treatment but data on their efficacy and adverse effects in Botswana is scarce. We determined the effect of amikacin on treatment outcomes and development of hearing loss in MDR-TB patients.

**Methods:**

Patients started on MDR-TB treatment between 2006 and 2012 were included. Multivariate analysis was used to determine the effect of amikacin on treatment outcomes and development of hearing loss.

**Results:**

437 MDR-TB patients were included, 288 (66%) of whom were HIV co-infected. 270 (62%) developed hearing loss, of whom 147 (54%) had audiometry. Of the 313 (72%) patients who completed treatment, 228 (73%) had a good outcome (cure or treatment completion). Good outcome was associated with longer amikacin treatment (adjusted OR [aOR] 1.13, 95% CI 1.06 - 1.21) and higher dosage (aOR 1.90, 95% CI 1.12 – 2.99). Longer amikacin duration (aOR 1.98, 95% CI 1.86 – 2.12) and higher dosage per weight per month (aOR 1.15, 95% CI 1.04 – 1.28) were associated with development of hearing loss. Amikacin treatment duration modified the effect of the dosage on the risk of hearing loss, increasing this risk as the duration increased.

**Conclusions:**

Amikacin was effective for MDR-TB treatment, but was associated with a high incidence of hearing loss especially in our study population. Total treatment duration and average monthly amikacin dose were associated with improved outcomes; however these were also associated with development of hearing loss.

## Background

Multidrug-resistant tuberculosis (MDR-TB) is caused by a *Mycobacterium tuberculosis* isolate that does not respond to isoniazid and rifampicin, the two most effective first-line antituberculous treatment (ATT) drugs. MDR-TB usually fails to respond to the conventional first-line ATT but is curable with second-line drugs. However, those drugs are limited in number and less effective. Fluoroquinolones and aminoglycosides (AGs) constitute the core of all currently recommended MDR-TB regimens [1–4]. Aminoglycosides are the cheapest and most accessible injectable antituberculous drugs. Therefore AGs are the injectable drugs of choice in most resource limited settings and also the ones recommended by the World Health Organization (WHO) guidelines [4, 5]. Similar to many other developing countries, Botswana has adopted a standardized MDR-TB regimen that included amikacin as the injectable drug of choice [5].

Aminoglycosides are potent antibacterials but also have substantial toxicity, potentially causing irreversible hearing loss. AG-induced hearing loss can occur sporadically, in a dose-dependent manner or based on genetic predisposition [6–9]. In resource-rich countries, the use of most AGs has been restricted to the treatment of gram-negative organisms. With this shift in the primary use of AGs, there was also a shift in the study of their toxicity. Over the last few decades, AG-related studies have focused on shorter treatment courses in hospitalized patients with gram-negative bacterial infections or on their use as synergistic agents [10–12]. In addition, most of those studies were performed in the pre-HIV era [13–17]. Thus, although many studies have described the toxic effects of AGs, the overall incidence of long term AG-associated hearing loss and risk factors predisposing patients to hearing loss remain disputed [18–20].

Given the lack of data, AGs dosages and treatment duration have been extrapolated from the experience in resource-rich settings to treatment of drug-susceptible TB. Specific data on AG-based regimens for the treatment of MDR-TB are limited and are particularly scarce in patients co-infected with MDR-TB and HIV [21, 22]. We analyzed the effects of amikacin-based regimens on the treatment outcomes of MDR-TB patients and the risk factors associated with the development of hearing loss.

## Methods

### Study design

Retrospective cohort.

### Study population

All MDR-TB patients 15 years of age and above who were started on MDR-TB treatment between January 01, 2006 and June 30, 2012 were eligible for the study [23–25]. We included all patients who had at least one baseline serum creatinine measurement (defined as creatinine measurement within 1 month prior to or one month after the start of amikacin) and one follow-up creatinine measurement (within the last 6 months prior to censoring). Patients who were deaf before initiation of MDR-TB treatment (defined by audiogram as an inability to detect sound at amplitude of 20 dB in a frequency range from 800 to 1,800 vibrations per second or by functionality as the absence of usable hearing) were excluded from the analysis.

### Setting and procedures

This study was conducted in Botswana, a sub-Saharan African country with HIV prevalence of 18% and a TB rate of 503/100,000 population [26, 27]. As per the national guidelines, all MDR-TB patients are referred to one of 5 specialized government TB clinics located around the country. These clinics served as the study sites. MDR-TB patients were placed on a standardized MDR-TB regimen whilst waiting for the second-line drug susceptibility test (DST) results [5]. The standardized MDR-TB regimen was composed of amikacin, levofloxacin, ethionamide, cycloserine, and pyrazinamide. Individualized regimens were provided after the second-line DST results were available [5]. The MDR-TB treatment was administered daily at the observation clinic. Amikacin dosage was calculated according to the WHO recommendations and adjusted for renal function [2]. Dosages raging between 15 and 25 milligram (mg) per kilogram (kg) intramuscularly (with a maximum dosage of 1000 mg per day) were administered. Amikacin was administered once daily, seven days per week, and changed to three times per week after culture conversion on patients who had hearing loss. The injection was then discontinued 4 months after culture conversion. At the time of analysis, audiometry (using GSI 61 audiometer) was not routine care for MDR-TB patients. High frequency audiometry was performed on some patients who reported hearing loss; follow-up on those with audiometry was performed every three months until 3 months after stopping amikacin.

Patients were treated for a minimum of 18 months after culture conversion. Clinical assessment and measurements, including weight, serum creatinine, sputum microscopy, cultures and DSTs were performed monthly [28–30]. An HIV test was performed on all patients with no documentation of infection [31].

### Data collection

Data was extracted from medical records and electronic databases at the TB clinics, Botswana National Tuberculosis Program, and Botswana National Tuberculosis Reference Laboratory. Data collected included patient demographics, semi-quantitative bacillary load by microscopy at the time of diagnosis (acid fast bacilli, [AFB]), extrapulmonary involvement, HIV status, and CD4+ cell count, along with the use of antiretroviral therapy (ART) in HIV-positive cases. All study participants (including those with evidence of concomitant extrapulmonary disease) had microbiological proof of pulmonary TB (PTB). TB was classified as unilateral or bilateral and cavitary or non-cavitary based on radiologic findings on chest X-rays. TB patients were also classified as having TB alone or PTB plus extrapulmonary involvement. Each month, mycobacterial culture results, DST results, number of antituberculosis drugs active at baseline, and average dose per kilogram of the injectable drug were collected.

### Outcome variables

We used two primary outcomes for our analyses:

- **Hearing loss.** We used two different definitions for hearing loss. Hearing loss confirmed by audiometry (our main outcome) was defined as an increase of 15 dB in pure tone threshold at two or more frequencies or an increase of more than 20 dB at one frequency. Among patients who did not undergo audiometry, clinical deafness was diagnosed by the treating physician functionally as absence of usable hearing acquired during or soon after (within 6 months after) amikacin treatment. In addition, we also used a composite outcome which combined all patients with audiometry-defined or clinically-defined hearing loss.
- **Treatment outcome.** We used the updated WHO definitions for treatment outcomes [32]. “Cure” was defined as MDR-TB patients who have completed treatment according to programme protocol without evidence of failure and had three or more consecutive negative cultures taken at least 30 days apart after the intensive phase. “Treatment completion” was MDR-TB patients who had completed treatment according to programme protocol without evidence of failure but with no record that three or more consecutive negative cultures were taken at least 30 days apart after the intensive phase. “Death” was defined as MDR-TB patients who die for any reason during the course of treatment. “Treatment failure” was defined as treatment terminated or need for permanent regimen change of at least two anti-TB drugs because of; lack of culture conversion (defined as two negative consecutive cultures taken at least 30 days apart; specimen collection date of the first negative culture was used as date of conversion) by the end of intensive phase, *or* bacteriological reversion (defined as two consecutive cultures taken at least 30 days apart, were found to be positive after initial conversion). Reversion was considered only when it occurred in the continuation phase after conversion to negative *or* if there was evidence of additional acquired resistance to fluoroquinolones or second-line injectable drugs. “Lost to follow-up” was used to describe MDR-TB patients whose treatment was interrupted for two or more consecutive months for any reason without medical approval [32]. For our main analyses, clinical outcome was defined as good (i.e., cure or completion of treatment) or poor (i.e., treatment failure, lost to follow-up or death).

### Statistical analysis

Categorical variables were summarized using frequencies and proportions, and continuous variables were summarized using mean values and medians. Differences in baseline characteristics of exposed and unexposed patients were assessed using chi-squared and t-tests or Wilcoxon rank-sum tests, as appropriate.

Our main exposures of interest were amikacin treatment duration (in months) and mean monthly amikacin dosage (per kg, per month). We performed two different analyses, for which we used different primary outcomes.

The first analysis aimed at identifying factors associated with the development of hearing loss. We performed three independent analyses using the above mentioned definitions of hearing loss (audiometry, clinical and audiometry plus clinical hearing loss). We used Pearson’s correlation coefficient to quantify the correlation between audiometry-defined and clinically-defined hearing loss. Patients who were still on treatment at the time of analysis (and did not have treatment outcomes) were excluded from these analyses.

Our data were not independent because of repeated measures on the same individuals over time. Thus, multilevel mixed-effect logistic regression models were used to calculate crude and adjusted odds ratios (aORs) and corresponding 95% confidence intervals (CIs) and to assess confounding. Mixed-effect logistic regression models enabled the correlations between repeated measures to be taken into account. We built these models to accommodate the correlation inherent in our data and account for clustering. A post estimation test, “Wald test” for simple and composite linear hypotheses about the parameters of a fitted model were computed to compare levels of multilevel variables. Pearson’s correlation test was also conducted to determine the correlation between cluster-level independent variables included in the models. Variance components were estimated using the restricted maximum likelihood method. We tested for presence of a statistical interaction between mean monthly amikacin dose per kilogram (Kg) and duration of amikacin treatment (in months) using a heterogeneity test.

Potential confounders included age, sex, prior TB history, baseline weight, and HIV. For analyses restricted to those with HIV, we also evaluated the CD4+ T cell count and use of anti-retroviral therapy (ART) at baseline as potential confounders. Potential confounders were considered actual confounders if their inclusion in the multivariable model changed the unadjusted ORs by 10% or more. We also determined if severity of TB disease was worse among those patients who developed hearing loss. However, adjusting for severity of disease could adjust for factors on the causal pathway between exposure (amikacin dose and time) and treatment outcome if the exposure affects risk of poor outcomes via worsening the severity of TB disease (e.g. higher dose of amikacin given to sicker patients, leading to renal failure). Factors indicating severity of disease included semi-quantitative bacillary load (by AFB microscopy: scanty, 1+, 2+ and 3+). Once our final model was selected, we assessed its reliability using a split-sample analysis. To develop the covariance model, we started with an unstructured covariance structure and then compared the fits of several other covariance structures using comparisons of likelihoods and information criteria. Finally, an unstructured covariance structure was selected because of substantial reduction in the likelihood when compared with other covariance structures.

The variables included in the “maximum model” were baseline weight, TB history, prior exposure to ATT, extent of radiological infiltrates (unilateral vs. bilateral), presence of cavitary lesions on the chest X-ray, and semi-quantitative bacillary load determined by microscopy. To account for potential differences in treatment that may affect outcomes, we adjusted for indicators of the appropriateness of treatment, including treatment duration with an injectable drug, the number of effective drugs used during the initial empirical therapy and the individualized therapy following the availability of second-line DST results. HIV infection, concomitant administration of ART and baseline CD4+ T cell count were also accounted for, where appropriate. Time-dependent variables included culture and DST results, monthly average number of TB drugs active against the MDR-TB isolate as indicated by prior culture, and monthly amikacin injectable dosage. Creatinine clearance was calculated using Cockcroft-Gault formula using monthly serum creatinine collected [33–36].

The second analysis aimed to determine the effect of amikacin over treatment outcomes. Treatment outcomes were first based on the WHO definitions [32] and then dichotomized as “poor” or “good”. Poor treatment outcome was defined as treatment failure, lost to follow-up, or death. Good treatment outcome was defined as bacteriological cure or completion of treatment. For this analysis, individuals were followed from MDR-TB treatment initiation date until they experienced a poor treatment outcome or were censored, which occurred if they remained on ATT at the end of observation or were lost to follow-up (>2 months since last visit) or at the end of observation (June 30, 2012), whichever came first. Collinearity was assessed by measuring variance inflation factors. Highly collinear variables were taken out from the model or, if they were considered important for the theoretical model, they were included one at a time.

### Ethics

This study was approved by the Human Research Development Committee at the Ministry of Health, Botswana, and the University of Pennsylvania Institutional Review Board.

## Results

### Cohort and study population

Of the 437 MDR-TB patients who met the inclusion criteria, 240 (55%) were male and 197 (45%) females. The median age was 38 years (interquartile range [IQR], 31-49). A total of 256 (59%) patients had previous streptomycin use, and 12 (3%) had previous amikacin use. The HIV status was available for all patients, and 288 (66%) were HIV-positive (Table 1). For those with no documented hearing loss, treatment was successful in 60 (36%) patients while 29 (17.4%) died during follow-up.

**Table 1:** **Demographic and clinical characteristics of patients initiated on MDR-TB treatment**

				Hearing loss by type of diagnosis	
Variables		All patients n = 437 n (%)	No hearing loss n = 167 (38.2%) n (%)	Hearing loss confirmed by audiogram n = 147 (33.6%) n (%)	Clinical diagnosis of hearing loss n = 123 (28.2%) n (%)
Sex	Female	197 (45.1)	71 (42.5)	63 (42.9)	63 (51.2)
	Male	240 (54.9)	96 (57.5)	84 (57.1)	60 (48.8)
Age category in years	15 – 19	28 (6.4)	19 (11.4)	6 (4.1)	3 (2.4)
	20 – 29	85 (19.5)	41 (24.6)	19 (12.9)	25 (20.3)
	30 – 39	127 (29.0)	40 (24.0)	47 (32.0)	40 (32.5)
	40 – 49	100 (22.9)	29 (17.4)	44 (29.9)	27 (22.0)
	50 – 59	65 (14.9)	25 (15.0)	23 (15.7)	17 (13.8)
	≥ 60	32 (7.3)	13 (7.8)	8 (5.4)	11 (8.9)
MDR-TB clinic	Clinic 1	101(23.0)	36 (21.6)	27 (18.4)	38 (30.9)
	Clinic 2	218 (50.0)	82 (49.1)	78 (53.1)	58 (47.1)
	Clinic 3	35(8.0)	9 (5.4)	14 (9.2)	12 (9.8)
	Clinic 4	40(9.2)	17 (10.2)	10 (6.8)	13 (10.6)
	Clinic 5	43(9.8)	23 (13.8)	18 (12.2)	2 (1.6)
History of prior TB treatment	Never treated for TB before	27 (6.2)	13 (7.8)	8 (5.4)	6 (4.9)
	New TB regimen	142 (32.5)	59 (35.3)	38 (25.9)	45 (36.6)
	Retreatment regimen	256 (58.6)	95 (56.9)	94 (64.0)	67 (54.5)
	*Treated for MDR-TB	12 (2.7)	0 (0.0)	7 (4.8)	5 (4.1)
Outcome	Completed	97 (22.2)	24 (14.4)	48 (32.7)	25 (20.3)
	Cured	131 (30.0)	36 (21.6)	69 (46.9)	26 (21.1)
	Death	53 (12.1)	29 (17.4)	10 (6.8)	14 (11.4)
	Lost to follow up	10 (2.3)	4 (2.4)	3 (2.1)	5 (4.1)
	Failure	22 (5)	7 (4.3)	5 (3.4)	8 (6.5)
	On treatment	124 (28.4)	67 (40.1)	12 (8.2)	45 (36.6)
HIV	HIV-uninfected	149 (34.1)	64 (38.3)	42 (28.6)	43 (35.0)
	HIV-infected	288 (65.9)	103 (61.7)	105 (71.4)	80 (65.0)
On anti-retroviral therapy	No	19 (7.0)	10 (9.7)	4 (3.8)	5 (6.2)
	Yes	267 (93.0)	93 (90.3)	101 (96.2)	73 (93.8)
CD + T 4 cell count cells/milliliter (mL) category	< 50 cells/mL	44 (17.0)	16 (15.6)	16 (15.2)	12 (15.0)
	50 – 199 cells/mL	52 (20.2)	17 (16.5)	21 (20.0)	14 (17.5)
	200 – 350 cells/mL	96 (37.2)	32 (31.0)	38 (36.2)	26 (32.5)
	> 350 cells/mL	66 (25.6)	38 (36.9)	30 (28.6)	28 (35.0)

*Previous history of MDR-TB treatment before enrolment in the study.

### Factors associated with amikacin-related hearing loss

Overall, hearing loss was diagnosed in 270 (62%) patients. Of them, 147 (54%) had audiogram confirmation. There was a perfect correlation between our definition of clinical deafness and severe hearing loss by audiometry (Pearson coefficient = 1.0) and no clustering was found. The diagnosis of hearing loss occurred at a median time of 170 days (IQR, 104-212 days) in those confirmed by audiogram and 167 days (IQR, 108-214 days) those without audiogram after amikacin initiation. Most patients (329, 75.5%) received the WHO recommended dosage range for their weight and renal function during the entire duration of treatment. Fifteen (3%) patients halted amikacin due to adverse effects other than hearing loss, and 316 (72%) completed the course. Eight (2%) patients died whilst on amikacin (Table 2).

**Table 2:** **The Characteristics of amikacin dose and duration in patients on MDR-TB treatment**

		Hearing loss by type of diagnosis	
	No hearing loss	Hearing loss confirmed by audiogram	Clinical diagnosis of hearing loss
Total patients, n (%)	167 (38.2%)	147 (33.6%)	123 (28.2%)
Baseline (initial amikacin dose) dose/kg(kilogram)	17.0 (9.3 – 22.9)	17.2 (15.4 – 18.9)	17.2 (15.3 – 18.8)
Mean daily dose of amikacin (dose/kg/month)	16.2 (14.1 – 18.3)	16.7 (15.3 – 18.9)	16.6 (14.9 – 18.8)
Number of patients in whom amikacin administration was changed from daily to 3 times per week	51 (30.5%)	131 (89.2%)	115 (93.5%)
Mean duration of daily amikacin treatment (days)	164 (2 – 429)	167 (105 – 210)	175 (126 – 213)
Mean duration of amikacin treatment given 3 times per week (days)	67 (2 – 336)	64 (41 – 105)	68 (42 – 108)
Overall duration of amikacin treatment (regardless of censoring; days)	189 (2 – 528)	195 (161 – 243)	200 (175 – 249)
Duration of amikacin treatment before censoring (days)	189 (42 – 632)	170 (104 – 212)	167 (108 – 214)

Our multivariate analyses showed that none of the demographic variables were associated with development of hearing loss. Similarly, HIV infection was not associated with increased risk of hearing loss. The most important risk factors for hearing loss were amikacin treatment duration (aOR 1.98; 95% CI 1.86-2.12, Table 3) and amikacin dosage (aOR 1.15; 95% CI 1.04-1.28, Table 3). The independent effect of a higher amikacin dosage on the risk of hearing loss was present even when dosages were within the recommended range for patients’ weight and renal function during the entire duration of treatment (data not shown). There was a strong positive interaction between amikacin treatment duration and dosage administered (Table 3).

**Table 3:** **Multivariate analysis of factors associated with risk of hearing loss among patients treated for MDR-TB**

		Hearing loss (diagnosed clinically and through audiometry) aOR (95% CI)	Hearing loss confirmed by audiometry aOR (95% CI)	Hearing loss diagnosed clinically aOR (95% CI)
Age category in years	15 – 29 years	1.00	1.00	1.00
	30 – 39 years	3.8 (1.46 – 9.88)	2.88 (0.96 – 8.63)	4.89 (1.26 – 18.97)
	40 – 49 years	5.21 (1.93 – 14.04)	4.49 (1.44 – 14.00)	5.57 (1.39 – 22.36)
	≥ 50 years	2.78 (0.83 – 9.34)	1.99 (0.46 – 7.87)	4.12 (0.83 – 20.51)
Sex	Female	1.00	1.00	1.00
	Male	0.72 (0.46 – 1.37)	0.78 (0.467 – 1.30)	0.64 (0.37 – 1.11)
HIV status	HIV infection	1.32 (0.83 – 2.12)	1.53 (0.89 – 2.62)	1.13 (0.62 – 2.06)
TB treatment history	Never treated for TB before	1.00	1.00	1.00
	New TB regimen	1.24 (0.51 – 3.04)	1.01 (0.38 – 3.02)	1.47 (0.46 – 4.66)
	Retreatment regimen	1.64 (0.69 – 3.91)	1.63 (0.60 – 4.36)	1.56 (0.51 – 4.78)
	*Treated for MDR–TB	Predicts outcome perfectly	Predicts outcome perfectly	Predicts outcome perfectly
Mean creatinine clearance per month in millilitre [mL]/minute	> 60 mL/minute	1.00	1.00	1.00
	40 – 60 mL/minute	1.48 (1.05 – 2.09)	1.07 (0.50 – 2.28)	1.27 (0.54 – 2.98)
	20 – 40 mL/minute	1.81 (0.87 – 3.75)	3.35 (1.07 – 10.53)	1.19 (0.29 – 4.82)
	< 20 mL/minute	1.51 (0.67 – 3.36)	1.52 (0.34 – 6.71)	1.18 (0.25 – 5.40)
^£^Duration of amikacin treatment in months		1.98 (1.86 – 2.12)	1.85 (0.94 – 3.99)	1.93 (0.89 – 3.97)
^¥^Mean dose of amikacin per kilogram per month		1.15 (1.04 – 1.28)	1.11 (1.00 – 1.23)	1.18 (1.04 – 1.33)
Interaction between the duration of amikacin treatment and amikacin dose		1.23 (1.11 – 1.35)		

*Previous history of MDR–TB treatment before enrolment in the study, ^¥^aOR of amikacin dose indicates the increasing risk of ototoxicity per mg/kg/month, ^£^aOR of duration of amikacin treatment indicates the risk of ototoxicity per month.

### Factors associated with good clinical outcomes

Our final multivariate model accounted for prior history of TB, prior TB treatment (for drug susceptible and MDR-TB), renal failure and HIV status. Demographic variables included in our models did not contribute to goodness of fit (Table 4). MDR-TB treatment and development of renal failure at any point during treatment were risk factors for poor treatment outcome. Longer duration (aOR 1.14, 95% CI 1.06 - 1.21) and a higher dosage of amikacin treatment (aOR 1.90, 95% CI 1.79 - 3.00) were associated with good outcome. Development of hearing loss during the treatment course was strongly associated with good outcome (aOR 3.29, 95% CI 1.77 – 6.10).

**Table 4:** **Multivariate analysis of factors associated with good clinical outcomes among patients treated for MDR-TB**

		Good treatment outcomes among all MDR TB patients aOR (95% CI)	Good treatment outcomes (excluding patients with hearing loss diagnosed clinically) aOR (95% CI)	Good treatment outcomes (excluding patients with hearing loss diagnosed by audiometry) aOR (95% CI)
Sex	Female	1.00	1.00	1.00
	Male	0.72 (0.4 –1.21)	0.71 (0.42 – 1.19)	0.72 (0.44 – 1.21)
HIV status	HIV infection	0.64 (0.37 –1.12)	0.70 (0.39 – 1.20)	0.66 (0.38 – 1.15)
TB treatment history	Never treated for TB before	1.00	1.00	1.00
	New TB regimen	0.93 (0.27 – 3.23)	0.71 (0.19 – 2.64)	0.75 (0.20 – 2.77)
	Retreatment regimen	0.60 (0.18 – 1.98)	0.48 (0.13 – 1.69)	0.48 (0.14 – 1.70)
	*Treated for MDR TB	0.16(0.09 – 0.67)	0.12 (0.02 – 0.70)	0.11 (0.02 – 0.57)
Renal failure at any point		0.61 (0.36 – 0.98)	0.46 (0.22 – 0.94)	0.52 (0.12 – 1.01)
Duration of amikacin treatment (in months)		1.13(1.06 – 1.21)	1.14 (1.06 – 1.21)	1.14 (1.05 – 1.22)
^¶^Mean dose of amikacin per kilogram per month		1.90 (1.12 – 2.99)	1.90 (1.79 – 3.00)	1.88 (1.18 – 3.99)

*History of MDR–TB treatment before enrolment in the study, ^¶^Minimum dose 13 mg/kg.

## Discussion

Our MDR-TB program achieved high rates of treatment success and our results support the inclusion of amikacin in the MDR-TB regimen. However, we also found that prolonged amikacin therapy and higher dosages per kg were associated with high incidence of hearing loss. Further, we found an interaction between dose and time of amikacin treatment. These data suggest that both good treatment outcomes and hearing loss are associated with higher amikacin dosages and longer administration durations highlighting one of the major issues that policy makers and clinicians face with regard to use of amikacin in MDR-TB patients: how can one balance the efficacy of treatment and the risk of deafness.

The bactericidal effect of AGs is concentration-dependent, meaning that increasing AG concentration kills an increasing proportion of mycobacteria at a higher rate. This property of AGs has been demonstrated *in vitro* for MDR-TB strains [37]. Our results support the conclusion that the bactericidal effect of AGs on MDR-TB *in vivo* is also concentration-dependent. We found that increasing amikacin dosages was associated with good treatment outcome. Our analysis also demonstrated that there were improved outcomes among MDR-TB patients treated with amikacin for longer periods of time. As for the beneficial effect of increasing amikacin dosage, we did not find duration of amikacin administration at which its beneficial effect plateaued. Currently, the WHO recommends using an injectable drug as part of any MDR-TB regimen for a minimum of 8 months [2]. However, data supporting this recommendation is sparse in the setting of prolonged therapy and, perhaps, in the absence of severe adverse effects longer courses of AGs might be indicated.

The incidence of hearing loss in our cohort, was close to 70% which is higher than what has been documented from other countries, despite that the majority (75%) of our patients were receiving an adequate dosage per weight and renal function as per the WHO guidelines [14, 38, 39]. Same as AG efficacy, adverse effects are both time and concentration dependent. Consistently in our study, development of hearing loss was independently associated with amikacin duration and dosage. In addition, these two risk factors showed an interaction with each other. In other words, the risk of hearing loss associated with amikacin treatment duration was different (increased) with increasing treatment doses. Although this interaction has been suggested by prior studies, to our knowledge, we are the first demonstrating it in the treatment of MDR-TB patients, where the recommended length of AG treatment may make this toxicity issue more clinically relevant.

Although the incidence of hearing loss in our cohort is perhaps the highest one reported to date, we believe that this incidence might be an underestimate. As per our definitions, we used highly specific criteria for the definition of hearing loss identified by audiometry. When audiograms were not available, hearing loss was defined by evidence of hearing loss at conversational level. From prior animal and human studies, we know that AGs initially cause irreversible high-frequency hearing loss by destroying outer hair cells while sparing inner hair cells [39–44]. This destruction gradually progresses into inner hair cells of the cochlea, and by the time this damaging effect becomes evident on a conventional pure-tone audiogram or at conversational level, high-frequency hearing has been already affected [17, 39]. Fully consistent with our findings, other clinical studies have shown that patients who lose the ability to hear a conversation at “whispering level” already have very advanced hearing loss according to their audiograms. Therefore, we are confident that patients who had evidence of hearing loss during the physical exam would also have hearing loss that could be detected by audiometry.

We did not find any association between hearing loss and HIV infection. Although our bivariate analysis showed an association between HIV infection and hearing loss, this association disappeared after adjusting for confounders. Thus, we believe that HIV infection by itself does not lead to a higher risk of developing hearing loss but is likely associated with a myriad of factors that by themselves or in combination increase the patient’s risk of AG-induced hearing loss. There were several limitations to this study. The therapeutic window of AGs is narrow. Therefore abnormally high-plasma or serum trough concentrations soon after starting therapy may have been missed, as the only parameters used to select the patient’s dose depended on weight and renal function in our population. We were also not able to obtain early audiograms for all our patients. Thus, it is highly likely that many patients already had some degree of hearing impairment at the time of the start of amikacin therapy, particularly those who had been on retreatment regimen. Due to limitations in our data, we were not able to analyse prior use of AGs. However, we found that history of MDR-TB treatment which could be a good surrogate for prior AG treatment. Similarly, given the lack of audiograms for some patients, we cannot confirm that the hearing loss we observed was sensorineural and that AGs were responsible for the hearing loss. We also depended on the diagnosis of hearing loss by physicians, which was not fully standardized and was performed, in most cases, only when hearing loss was severe. Medications other than ART, which may have been ototoxic or protective, were not accounted for. Our analyses included all patients at risk for ototoxicity. Our implicit assumption was that all patients have an equal risk of development of ototoxicity at any point in time. Since patients on treatment are at risk for the development of the outcome, they were also included in the analyses. We acknowledge that this assumption might not be absolutely true (given potential differences in susceptibility and other confounders). However, after adjustment for confounders and particularly time in the study, we believe it provides the most robust analyses to look into the relationship between AGs and ototoxicity.

## Conclusions

MDR-TB will continue to be a global health problem for many years to come. Although new and promising drugs are in the pipeline, it seems likely that AG-based regimens will continue to be the standard of care in many resource-limited settings. Therefore, prospective research studies to determine the best balance between the benefits and adverse effects of these drugs are critical. Future studies must investigate inexpensive, easy-to implement interventions to identify early AG-related hearing loss.
